# Oil Sands Development: A Health Risk Worth Taking?

**DOI:** 10.1289/ehp.117-a150

**Published:** 2009-04

**Authors:** David J. Tenenbaum

As traditional petroleum supplies dwindled and prices soared over the past few years, oil companies have shifted their attention to oil sands, a mix of sand, water, and a heavy, viscous hydrocarbon called bitumen that can be converted to oil. With the plunge in oil prices in fall 2008, many producers began canceling or postponing plans to expand oil sands development projects, but this turn of events could yet reverse, as Canada’s vast oil sands deposits are lauded as a secure source of imported oil for the United States. At the same time, however, oil sands present troubling questions in terms of the environmental health effects associated with their development.

## Raw Resource

Oil sands are found in about 70 countries. Alberta, Canada, is home to the largest known oil sands deposits, underlying about 140,000 square kilometers of boreal forest. In the 2006 report *Alberta’s Energy Reserves 2005 and Supply Outlook 2006–2015*, the entity then known as the Alberta Energy and Utilities Board estimated the amount of recoverable oil in Canada’s oil sands at 175 billion barrels, second only to Saudi Arabia’s reserves (which consist largely of conventional oil).

Other major deposits are located in Venezuela and Utah. According to figures cited by Argonne National Laboratory, the Utah deposits, if developed, could yield 12–19 billion barrels of oil. However, says Philip Smith, a professor of chemical engineering and director of the Institute for Clean and Secure Energy at the University of Utah, the Utah bitumen cannot be recovered using the water-intensive technologies pursued elsewhere for one simple reason: “In Utah, we just do not have that kind of water.”

Over the past five years, production at Canada’s oil sands has reached about 1.3 million barrels per day, more than 1% of global oil production, according to the Canadian Association of Petroleum Producers (CAPP). This constitutes the bulk of the 1.9 million barrels Canada exported to the United States each day in 2008, an amount equal to 12% of U.S. total petroleum consumption, says Greg Stringham, vice president for oil sands at CAPP. In *North American Oil Sands: History of Development, Prospects for the Future*, a report last updated in January 2008, the Congressional Research Service estimated production would soar to 2.8 million barrels per day by 2015.

Shallow deposits containing about 8–20% of Alberta’s oil sands (depending on the estimate) are surface mined using giant shovels and enormous trucks. Deeper deposits more than 75–80 meters underground are accessed using methods such as steam-assisted gravity drainage, in which steam is used to heat the bitumen so it becomes fluid enough to be pumped to the surface. This is known as *in situ* production.

Unprocessed oil sands contain 3–18% bitumen by weight, along with 2–10% water and 80–85% mineral matter (sand, clay, etc.). Bitumen is composed chiefly of polycyclic aromatic hydrocarbons (PAHs), sulfur, lead, mercury, arsenic, nickel, vanadium, chromium, and selenium. It contains far more carbon and far less hydrogen than conventional crude oil; mixed with crushed stone, bitumen forms asphalt pavement. Once the bitumen is separated from the ore, it is “upgraded” through the addition of hydrogen and the subtraction of carbon, and natural gas is added to enable the material to be pumped to a refinery for processing. The remaining water and solids, including a small amount of unex-tracted bitumen, are discharged into vast tailings ponds.

## Tailings: A Threat to Water Quality

A good deal of the controversy about oil sands development centers around those tailings ponds, which cover more than 130 square kilometers in northern Alberta, according to the 2008 report *11 Million Litres a Day: The Tar Sands’ Leaking Legacy* from Canada’s Environmental Defence. Some large tailings ponds are separated by earthen dikes from the Athabasca River, which joins the Mackenzie River to form the major watershed of Northwest Canada. The water in these ponds often contains arsenic, mercury, PAHs, and other toxics found in the bitumen.

Oil sands operators maintain inter ceptor ditches and wells to catch leakage from the tailings ponds, but the Environmental Defence report calculated that 11 million liters of contaminated wastewater nevertheless escapes each day. This rate was based on data from oil companies’ environmental impact assessments; for ponds for which there were no publicly available data, the authors calculated an average seepage rate using industry-reported figures.

Opponents of oil sands development are concerned about the potential for adverse health effects if the leaking wastewater contaminates drinking water supplies. George Poitras, former chief of the Mikisew Cree First Nation, says chemicals leaking from tailings ponds “affect anybody or anything that relies on water as a source of drinking or a place to live in [including fish, moose, and birds]. The majority of our people rely on the traditional diets, on moose.”

Ecologist Kevin P. Timoney of Treeline Ecological Research believes the 11 million liters/day estimate is conservative; the actual rate, he says, is probably much greater. In *A Study of Water and Sediment Quality as Related to Public Health Issues, Fort Chipewyan, Alberta*, published in November 2007, Timoney described his analysis of published data on water and sediment quality indicators at the titular community, which is located at the northernmost edge of the Athabasca oil sands. He noted that Fort Chipewyan lies within a depositional basin in which metals and other contaminants tend to accumulate in fine-textured sediments. Concentrations of arsenic, mercury, and PAHs are especially high in water and sediment, and many other metals (including cadmium, chromium, cobalt, and lead) and agricultural chemicals also are present.

Timoney’s analysis further noted that studies of local fish have shown that all the walleye and female whitefish and almost all the male whitefish tested exceeded U.S. guidelines for mercury consumption. Although treated local water appeared safe, untreated water in Lake Athabasca had levels of arsenic, total mercury, and PAHs sufficient to pose a threat to wildlife or humans.

Glen Van Der Kraak, a professor in the Department of Integrative Biology at the University of Guelph, Ontario, says studies of fish exposed to oil sands wastewater consistently find endocrine disruption and impairments of reproductive physiology. For example, in research published in the 1 May 2008 issue of *Aquatic Toxicology*, Van Der Kraak and colleagues found that goldfish exposed to wastewater from tailings ponds had dramatically lower plasma levels of testosterone and 17β-estradiol than control fish. The prime suspect behind these effects, says Van Der Kraak, is naphthenic acids, compounds that are often present in tailings pond water.

John O’Connor, a doctor who practiced in Fort Chipewyan between 2002 and 2007, first raised the alarm about human cases of cholangiocarcinoma, reporting six possible cases in this community of about 900. This rare cancer of the bile duct typically strikes about 2 in 100,000 people. In Alberta, the incidence of cholangiocarcinoma has increased progressively over the past 30 years, and rates are 2–3 times higher in First Nations communities compared with non–First Nations populations.

In August 2007 a working group was convened to support the Alberta Cancer Board in performing a cluster investigation using guidelines from the U.S. Centers for Disease Control and Prevention. The number of observed cancer cases in the community, as determined through the Alberta Cancer Registry, was compared with the number of cases expected over a 12-year period. Expected cases were determined by applying yearly Alberta rates to the Fort Chipewyan population, taking into account the size and composition of the population.

In the February 2009 report *Cancer Incidence in Fort Chipewyan, Alberta, 1995–2006*, the group reported that only two of the cases in Fort Chipewyan were confirmed as cholangiocarcinoma. A third case was not a cancer, and the remainder were confirmed to be other cancers. Given these numbers, the incidence of cholangio-carcinoma fell within the expected range. However, the study found higher-than-expected numbers of cancers of the blood and lymphatic system, biliary tract, and soft tissue (all statistically significant findings), as well as all cancers combined (51 observed versus 39 expected cases—a finding deemed to be of borderline statistical significance). Lung cancers as a whole were within the expected range, but when women were looked at separately, the number of cases was 3.5 times higher than expected.

The study was not designed to determine the cause of any of the cancers observed, and because of the small population size and limited number of cases, the working group cautioned that the findings could be due to chance and/or increased detection. Given that the numbers rose in the latter half of the 12-year study period, however, the group wrote that closer monitoring of cancer occurrences in Fort Chipewyan will be justified in the coming years and that future studies should track a cohort of residents who have lived in the area within the past 20–30 years.

The authors also noted that a 2006 analysis of the health status of Fort Chipewyan residents showed that residents have elevated prevalence rates of diabetes, hypertension, renal failure, and lupus. All these diseases have been linked with one or more of the toxics commonly found in tailings pond water. The working group suggested that, in order to examine risks for cancer and other chronic diseases, assessment of the overall health status and risk factor profile of Fort Chipewyan residents would be needed. Future studies should also evaluate the occupational history and employment-related migration pattern of the cancer patients in the community, because many of the Fort Chipewyan residents work or had worked in the oil sands or uranium industries. As the authors pointed out, “Previous studies of cancer risk and occupational exposure have suggested increased risk of leukemia and lung cancer in oil field workers, and increased risk of leukemia, lung cancer, and cancers in gallbladder and extrahepatic bile ducts in uranium miners.”

## Long-Term Restoration Challenges

Although the authors of *Cancer Incidence in Fort Chipewyan* avoided assigning a cause for the cancers they observed, many critics of oil sands development believe it is only a matter of time before a link is established with tailings pond leakage. Moreover, in his 2007 report, Timoney asserted that abandoned tailings ponds could pose a major health threat to surrounding communities for years to come. “While a mine is in operation, monitoring and pumping of tailing pond leaks is continuous,” he wrote. “No one knows what will happen when a mine has exhausted a site, shuts down its operation, and leaves. Tailings pond abandonment is an unproven technology whose success is predicated on modeling rather than real world experience. . . . The [Alberta oil sands formation] is known to be porous with active subsurface water movements. Billions of cubic meters of contaminated water soon will be sitting untended, with no active pumping, in abandoned ponds adjacent to the Athabasca River.”

The challenges of restoring the tailings ponds and other elements of development sites have been underestimated, says E.A. Johnson, a professor of biological sciences at the University of Calgary and co-author of a report on the science behind reclamation in the oil sands published in *The Year in Ecology and Conservation Biology 2008*. “Restorations are usually small projects, a few hectares in size, but now we are confronted with whole landscapes in which the reconstruction must start with the central template, the groundwater, and then the soil. . . . We are going to have to reconstruct the drainage, the ground-water flow, and these are things about which we have little knowledge. It is not clear to me that everybody understands how complicated this is.”

Many years are needed to evaluate a restoration, Johnson adds. “Tradition ally, even in small restoration projects, it takes much longer than anyone imagines, especially for the monitoring. This calls for a 40-year attention span or more, and it will be hard to keep that going.”

According to *Alberta’s Oil Sands. Resourceful. Responsible*, a 2008 publication from the government of Alberta, as of March 2008 approximately 65 square kilometers of land were in the process of being reclaimed, meaning the land would “be able to support a range of activities similar to its previous use before oil sands development.” However, the province has certified only 104 hectares (at a facility run by major producer Syncrude) as restored.

Both opponents and proponents of oil sands development agree that liquid tailings are a problem. “We need to prohibit the creation of liquid tailings that require these tailings ponds,” says Simon Dyer, oil sands program director at the nonprofit Pembina Institute in Calgary, Alberta. “There are new technologies that are close to commercialization that would not require the creation of liquid tailings, but there is no incentive for companies to implement these when the government is willing to approve their projects.”

“The ultimate goal is dry tailings,” agrees Stringham, who notes that the industry is, in fact, working on near-term solutions, such as injecting carbon dioxide (CO_2_) into tailings so the clay can settle more quickly, allowing the water to be drawn off and reused.

## A Carbon-Intense Industry

The carbon intensity of oil sands development poses other environmental health questions. The extraction and refining of oil sands produces 30–70% more greenhouse gas emissions than conventional oil production, according to estimates by Alex Farrell and Adam Brandt published in the October 2007 issue of *Climatic Change*. If the greenhouse gas impact of oil sands is calculated to include the CO_2_ released when the fuel is burned, the discrepancy drops to 10–30%, says Aimee Curtright, an analyst at the RAND Corporation and coauthor of the 2008 report *Unconventional Fossil-Based Fuels: Economic and Environmental Trade-Offs*. “For both [conventional and unconventional] oil, most CO_2_ release occurs in burning; the smaller portion of greenhouse gases is related to the production process,” she says.

The primary global impact of oil sands comes through the release of greenhouse gases created when about 800 million cubic feet of natural gas (approximately 10% of Canada’s total natural gas consumption) is burned daily to create heat for extraction and upgrading, says Stringham. In the 2006 report *The Canadian Oil Sands in the Context of the Global Energy Demand,* Eddy Isaacs, director of the Alberta Energy Research Institute, wrote that 176 cubic meters of natural gas are required to liquefy, extract, and purify each cubic meter of bitumen produced.

According to the 2008 Congressional Research Service report, the government of Canada expects that by 2010 the oil sands will produce half of Canada’s growth in greenhouse gas emissions and 8% of the country’s total greenhouse gas emissions. The longer-term picture is even more striking, says Dyer. “Even under government predictions, oil sands emissions will triple by 2020. This is inconsistent with meaningful action on climate change. [Oil] sands . . . are almost single-handedly taking us in the opposite direction of [the Kyoto Protocol].”

Chris Bourdeau, a spokesperson for Alberta Environment, says this is an unfair characterization of the impact of oil sands. Oil sands produced 33 metric megatons (Mt) of greenhouse gas emissions in 2006, he says. Canada’s Kyoto commitment is to reduce emissions to 6% below 1990 levels. Canadian emissions in 1990 were 594 Mt, whereas emissions in 2006 were 721 Mt. “Canada is 163 Mt over its Kyoto target,” he says. “Oil sands, with 33 Mt of total emissions, are not single-handedly taking the country in the opposite direction—there are many factors.”

That said, the oil sands industry is being prodded to reduce energy use by Alberta’s Specified Gas Emitters Regulation, which requires oil sands operators and other industries that release more than 100,000 metric tons of CO_2_ equivalent per year to reduce their “emissions intensity”—or CO_2_ equivalents per unit of product—by 12% compared with a baseline measured during 2003–2005. “Alberta is the only jurisdiction in North America to have a regulatory system in place that creates mandatory emission reductions,” says Bourdeau.

Industries that cannot make the reduction can buy offsets, such as paying for reforestation inside Alberta or contributing Can$15 per metric ton of CO_2_ equivalent to the province’s Climate Change and Emissions Management Fund. Bourdeau says this program, in its first half-year of operation, resulted in a total reduction of 2.6 million metric tons of CO_2_ equivalent. Although the 12% reduction is a one-time cut, companies must pay annually for offsets or the technology fund if they cannot make the reduction. [For more information on carbon offsets, see “Carbon Offsets: Growing Pains in a Growing Market,” *EHP* 117:A62–A68 (2009).]

Carbon capture and storage (CCS), in which CO_2_ would be transferred to deep underground storage, is touted by oil sands advocates as the ultimate solution to greenhouse gases releases. In April 2008 the government of Alberta launched a council, led by former Syncrude president Jim Carter, to develop a roadmap for broad-scale implemention of CCS. In July 2008 the government committed Can$2 billion to support construction of “high-impact” CCS facilities starting this spring in the expectation that overall greenhouse gas emissions will be reduced by 5 million metric tons per year by 2015. However, although many experts believe CCS is viable in theory, it is largely untested on the scale proposed by the oil sands industry. [For more information on CCS, see “Carbon Capture and Storage: Blue-Sky Technology or Just Blowing Smoke?” *EHP* 115:A538–A545 (2007).]

## Uncertain Future

With President Obama now in office, all sides in the oil sands issue are pondering the schedule and details on any upcoming restrictions on greenhouse gases and what those will mean for oil sands development. One possibility is that the United States will restrict import of fuels that entail extra releases of greenhouse gases.

But a complete halt to oil sands extraction is unlikely, and the price of oil could easily start to rise again. Instead of advocating a halt, most critics of oil sands development favor simply slowing or stopping expansion. “First, we want a moratorium on any new development, to stop granting new permits, new leases, and new *in situ* development sites,” says Susan Casey-Lefkowitz, director of the Canada program at the Natural Resources Defense Council. “We think the governments in Alberta and Canada need to take stock of what has already happened to the land, the environment, and the people who live there, and try to remedy some of that harm. If they go forward, they need to figure out a way to do it that is environmentally sustainable.”

## Figures and Tables

**Figure:**
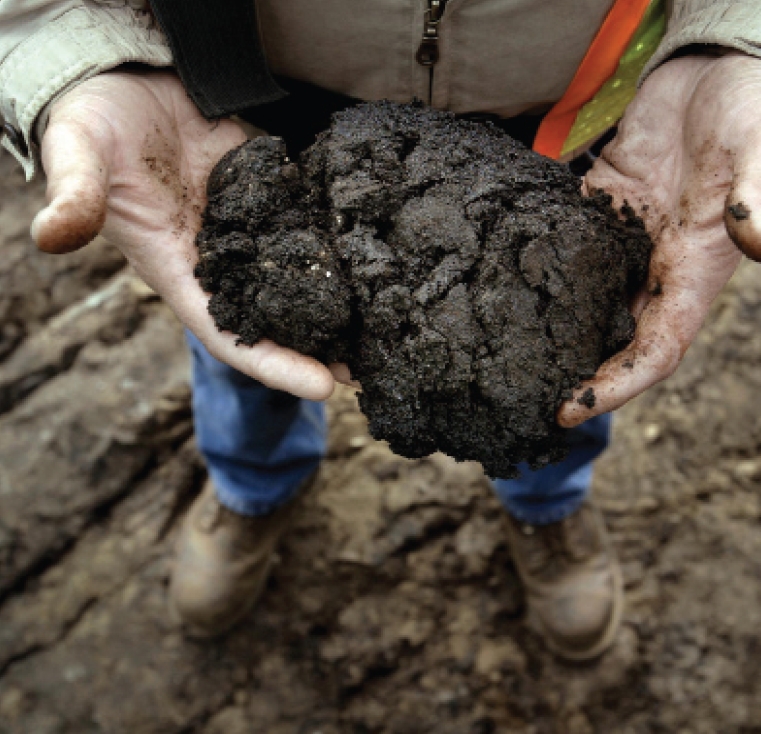
Bitumen, a tar-like hydrocarbon that can be converted to oil, is the key to oil sands’ value. Canada’s oil sands hold an estimated 175 billion barrels of recoverable oil, but realizing that potential is a costly undertaking.

**Figure:**
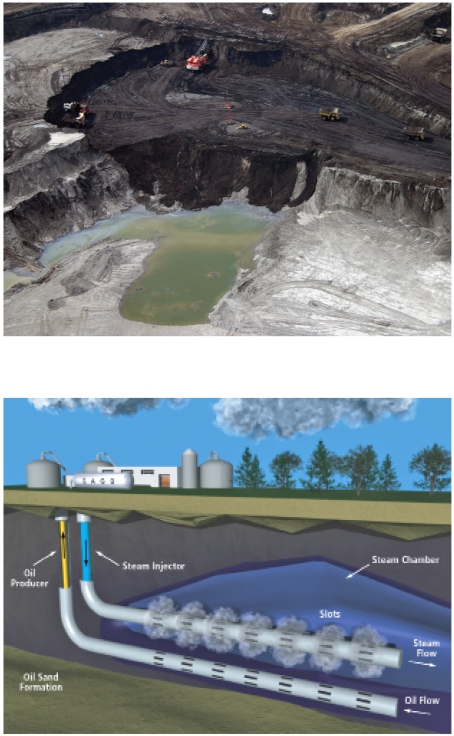
At surface mining facilities (above), trucks carrying hundreds of tons each transport ore to processing facilities. However, most of Alberta’s oil sand deposits lie deep underground, and the bitumen must be pumped to the surface using techniques such as steam-assisted gravity drainage (below).

**Figure:**
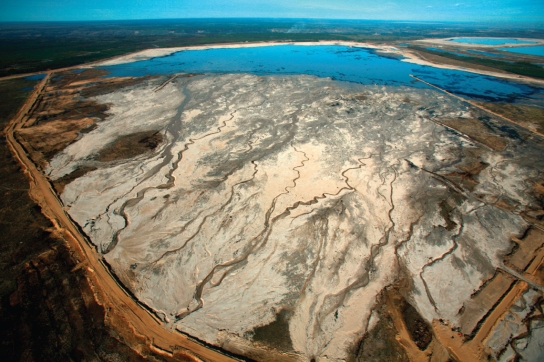
Aerial view of a tailings pond north of Fort McMurray, Alberta, Canada. One of the chief human health concerns associated with oil sands development is the leakage of contaminated wastewater into drinking water supplies.

**Figure:**
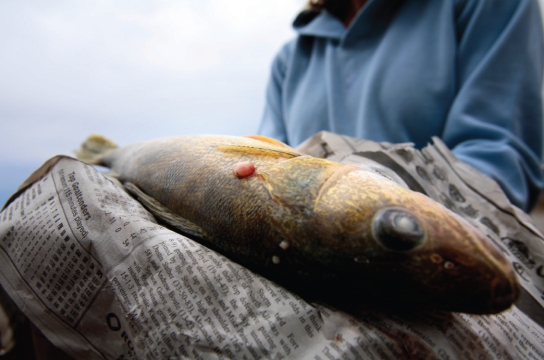
Scientists and local subsistence fishers have observed cancerous tumors on whitefish caught near Fort Chipewyan, a community on the northern edge of the Athabasca oil sands. A 2009 study reported a higher-than-expected number of human cancers in the community but was not designed to determine the cause of those cancers.

**Figure:**
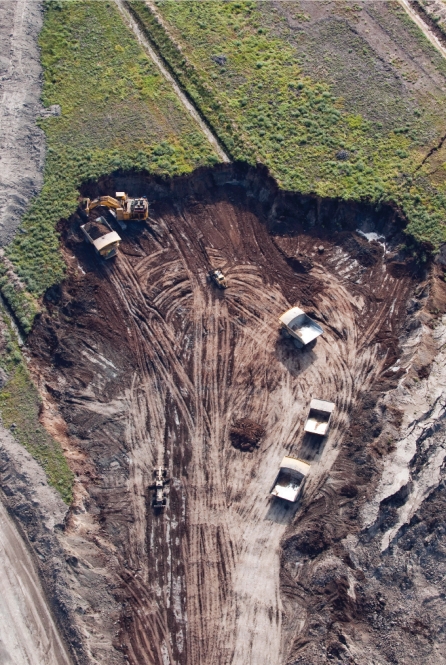
For 2005 Environment Canada estimated that industry and resource extraction accounted for 8% of deforestation in Canada, affecting less than 0.02% of the country’s forests. Although deforestation can have adverse ecologic consequences, the greater greenhouse gas impact of oil sands development comes from the energy used to extract and refine the bitumen.

